# A novel bedside swallowed optical sensor for detection of upper GI bleeding

**DOI:** 10.1016/j.vgie.2022.08.018

**Published:** 2022-09-28

**Authors:** Tala Mahmoud, Mohammad Alqaisieh, Karl Akiki, Kristin Lescalleet, Andrew C. Storm

**Affiliations:** Division of Gastroenterology and Hepatology, Mayo Clinic, Rochester, Minnesota

**Keywords:** SOS, swallowed optical sensor, UGIB, upper GI bleeding

## Abstract

Video 1 A novel bedside swallowed optical sensor for detection of upper GI bleeding.

A novel bedside swallowed optical sensor for detection of upper GI bleeding.

## Introduction

Gastrointestinal bleeding is a common emergent condition, accounting for 7% to 8% of acute medical admissions. In the United States, upper GI bleeding (UGIB) leads to an average of 300,000 admissions per year and has a mortality rate that ranges from 2% to 15%.^1^^,^^2^ EGD represents the mainstay diagnostic tool for suspected UGIB. However, timely EGD can be a challenging resource to access during non-business hours. A novel bleeding sensor system (PillSense; EnteraSense, Galway, Ireland) was developed to facilitate triage of patients with suspected UGIB. The swallowed optical sensor (SOS) capsule is not yet Food and Drug Administration approved and is limited to investigational use only in the United States. The first in-human trials of the SOS system demonstrated the safety and accuracy of this minimally invasive novel method in detection of UGIB.^3^^,^^4^

**Figure 1:**
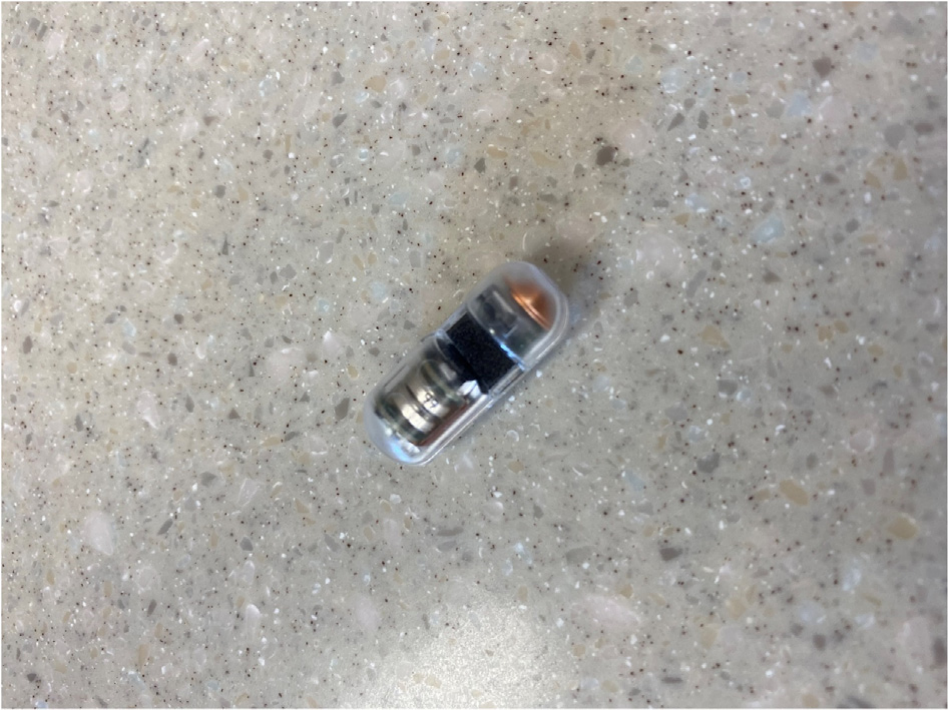
The disposable swallowed optical sensor capsule.

**Figure 2:**
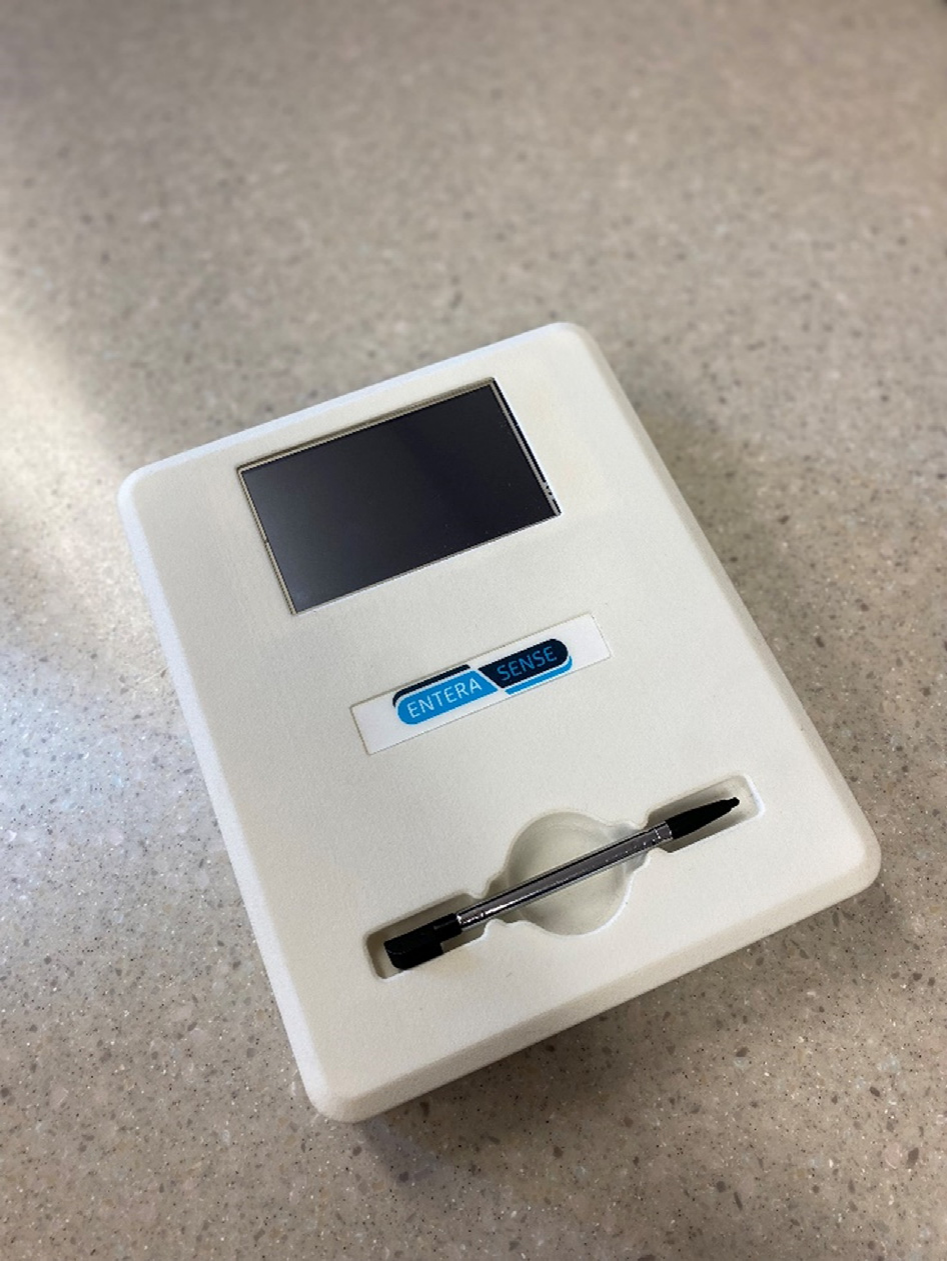
The external receiver.

The bleeding sensor system consists of a disposable SOS capsule (Fig. 1) and an external receiver (Fig. 2). The SOS capsule is a minimally invasive, single-use device designed to detect blood in the stomach. Detection of blood in the stomach is used for evaluating the presence of UGIB. The SOS capsule features an optical sensor that detects the presence of blood by measuring the absorption of multiple wavelengths of light. The SOS capsule was calibrated such that a spectrum of wavelength ranging from red blood through older coffee-ground colored material will be interpreted by the device as blood being present. The data are then wirelessly transmitted to the external receiver and processed by an algorithm to determine if blood is present. No endoscopic images are transmitted via the capsule. The receiver interprets the data and displays a result message of “Blood detected” or “No blood detected.” The capsule is designed to withstand the mechanical forces and chemical environment of the digestive system and thus will make its way through the GI tract and is then passed naturally from the body. The step-by-step instructions for usage of the bleeding sensor system are described in this video (Video 1, available online at www.giejournal.org).

## Case Presentations

**Figure 3:**
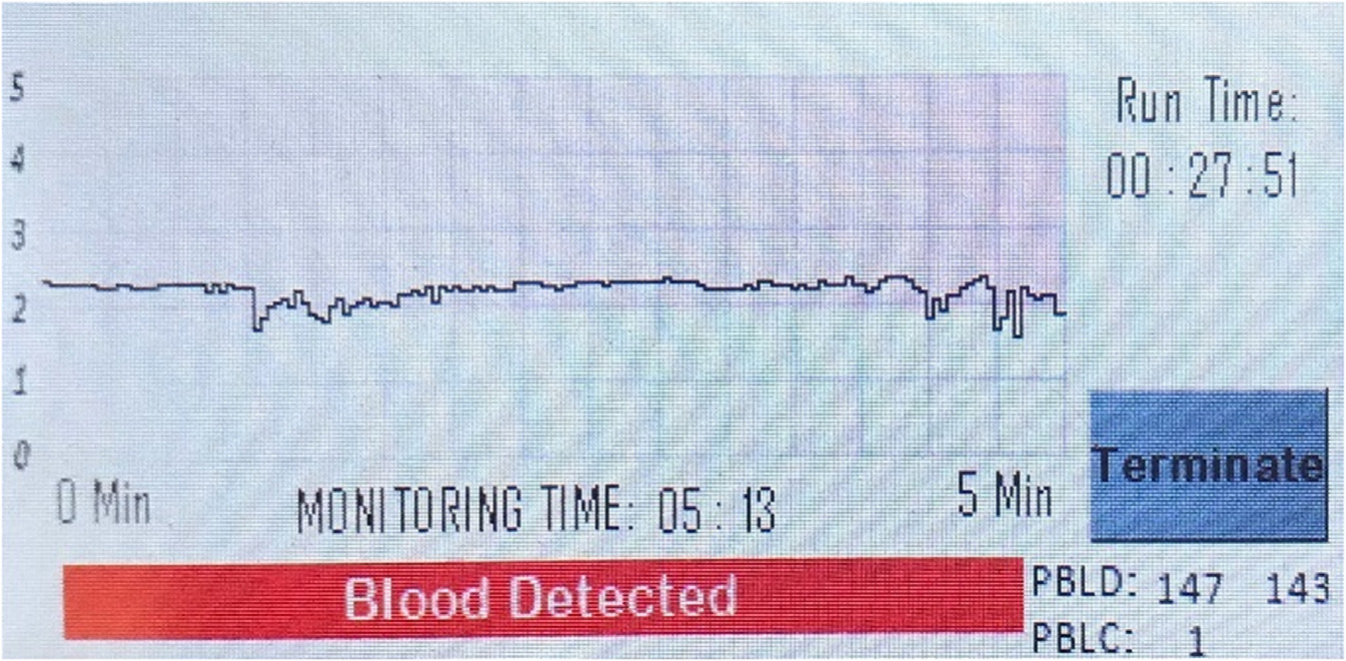
The external receiver indicating presence of blood.

**Figure 4:**
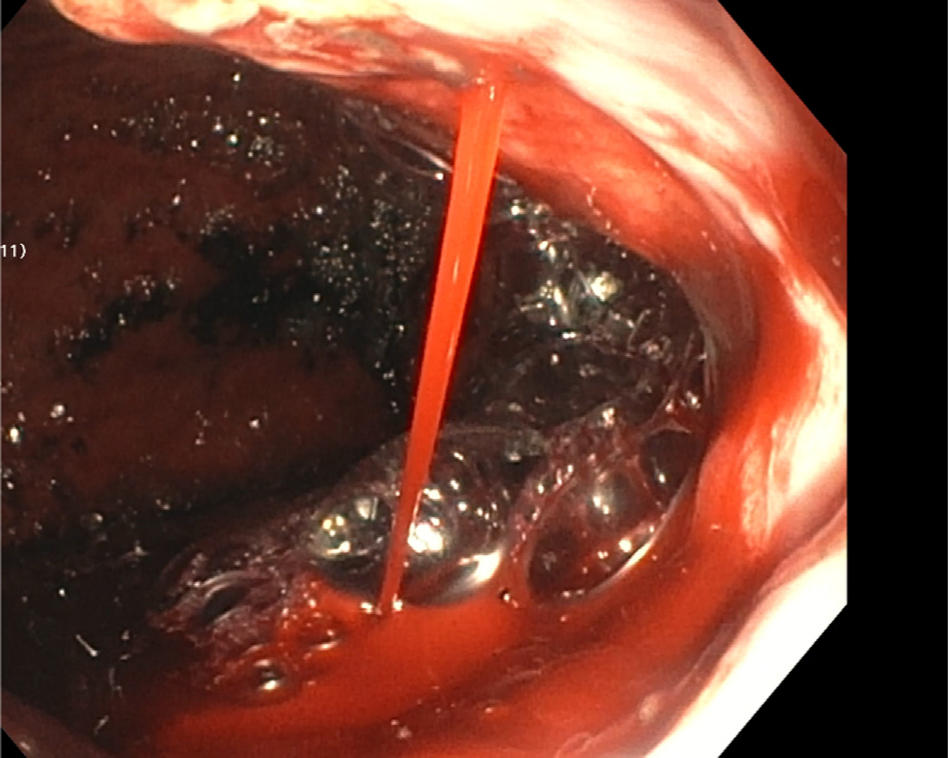
An active spurting varix at the gastroesophageal junction seen on EGD.

**Figure 5:**
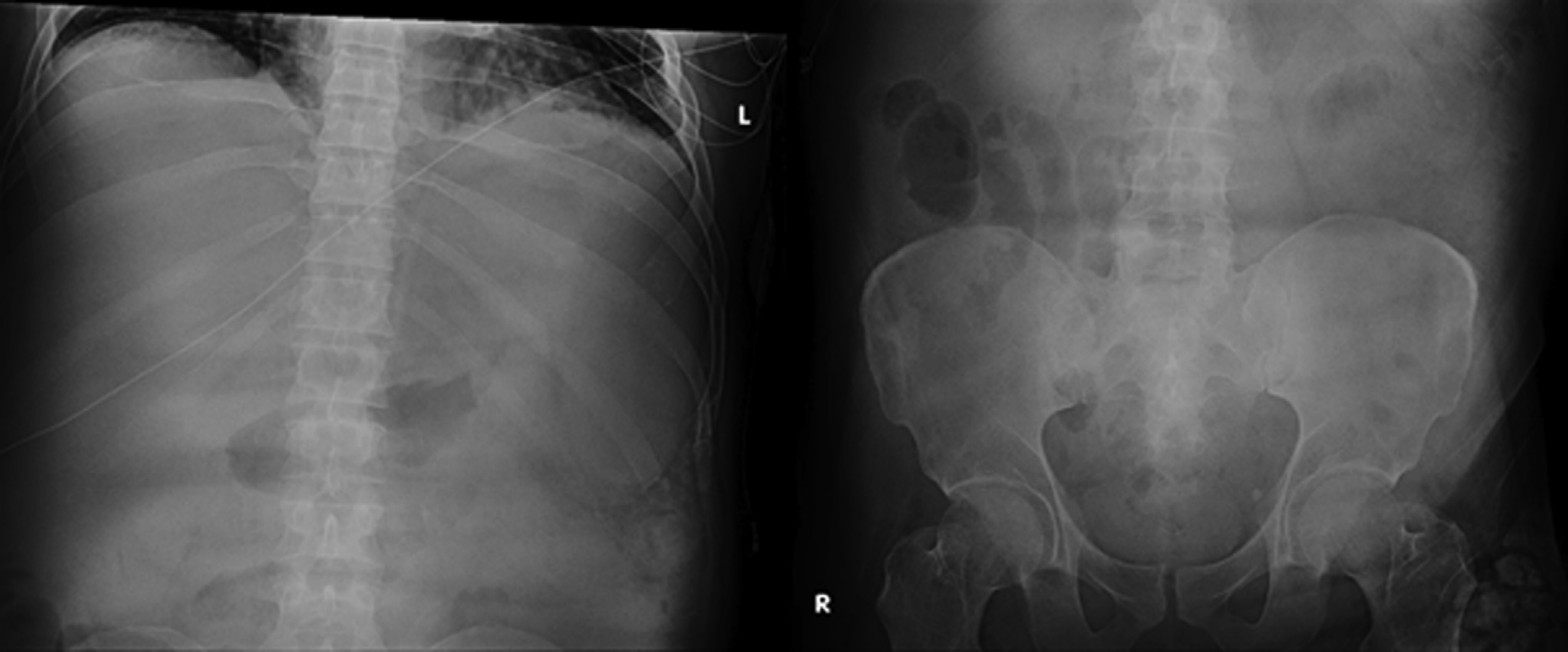
A follow-up abdominal radiograph confirming swallowed optical sensor capsule excretion.

In case 1, a 38-year-old male patient with a history of recurrent upper GI bleeding presented to the emergency department for hematemesis and coffee-ground emesis. The patient was hemodynamically stable. The external receiver indicated that blood was detected by the SOS capsule, and total monitoring time was 5 minutes and 13 seconds (Fig. 3). The patient underwent an EGD after 1.5 hours of capsule administration, which showed large varices in the lower third of the esophagus and an active spurting varix at the gastroesophageal junction that was actively pouring red blood into the stomach (Fig. 4). A total of 6 bands were successfully placed. A follow-up abdominal radiograph performed 3 days after capsule administration confirmed the SOS capsule had been excreted from the body (Fig. 5).

**Figure 6:**
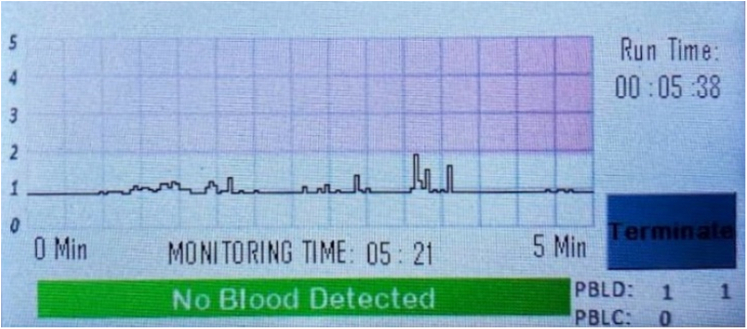
The external receiver indicating absence of blood.

**Figure 7:**
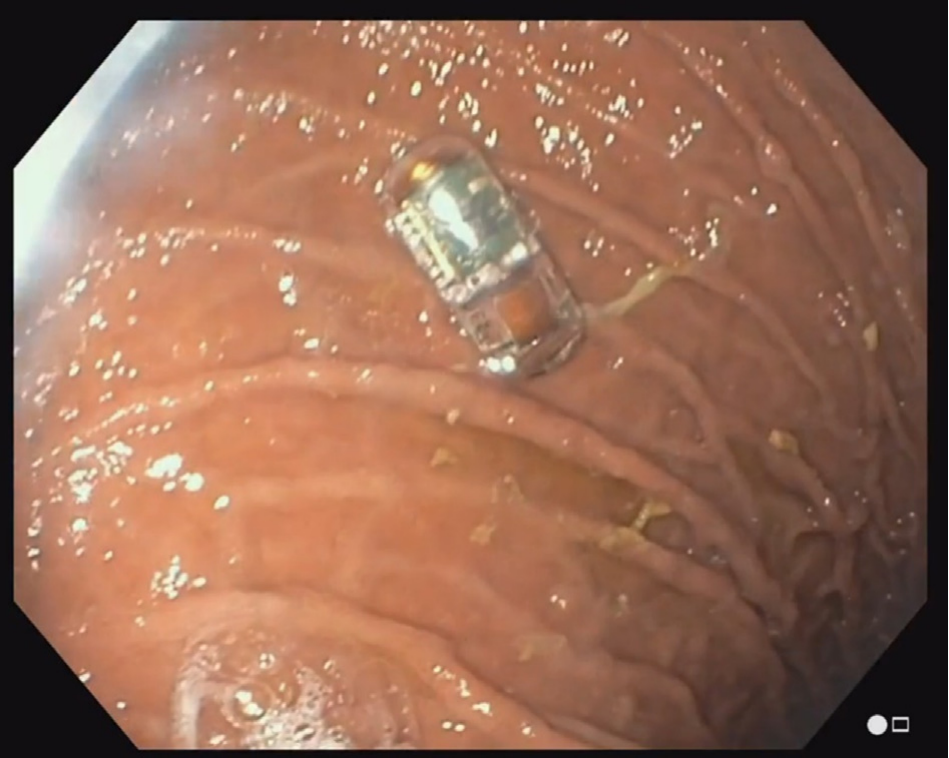
The swallowed optical sensor capsule visualized in the stomach on EGD.

**Figure 8:**
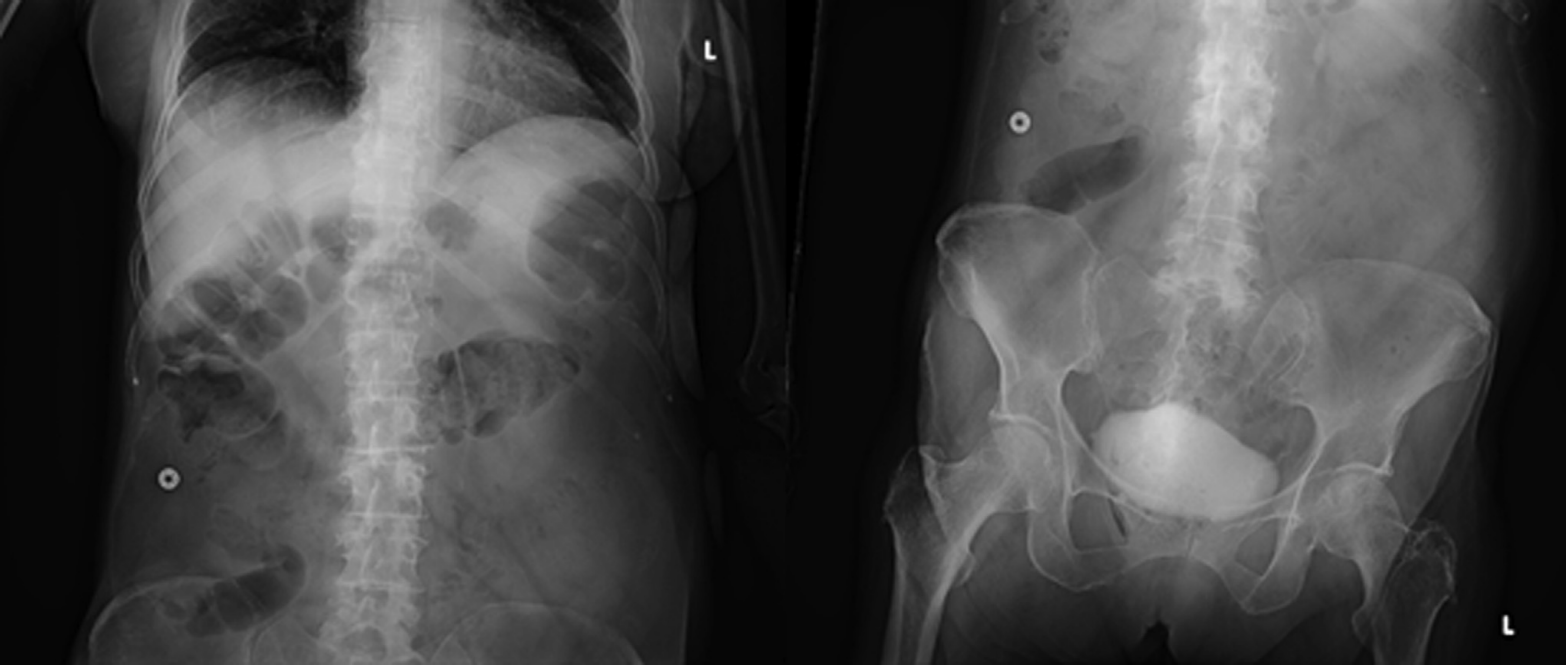
A follow-up abdominal radiograph confirming swallowed optical sensor capsule excretion.

In case 2, a 69-year-old female patient with a 2-week history of melena and progressive weakness in the setting of severe anemia presented to our department. The patient was on aspirin and dipyridamole for history of cerebrovascular disease with stroke but denied any previous episodes of GI bleeding. The patient was hemodynamically stable. The external receiver indicated that no blood was detected by the SOS capsule (Fig. 6). The patient underwent an EGD 30 minutes later, and the capsule was visualized in the stomach (Fig. 7). The EGD showed erosive gastropathy and non-bleeding duodenal ulcers with no bleeding or stigmata of recent bleeding. A follow-up abdominal radiograph performed 6 days later confirmed that the capsule had been excreted from the body (Fig. 8).

## Conclusion

The bleeding sensor system is simple and easy to use, without any learning curve required, and is designed to provide results in real time. The overall investigation from the capsule activation and ingestion until the result message is displayed takes less than 10 minutes.

## Disclosure

Dr Storm is a consultant for Apollo Endosurgery, ERBE, GI Dynamics, and 10.13039/100009734Olympus and received research grant support from Apollo Endosurgery, 10.13039/100008497Boston Scientific, Endo-TAGSS, Endogenex, and Enterasense. All other authors disclosed no financial relationships.

## Supplementary data

Video 1 A novel bedside swallowed optical sensor for detection of upper GI bleeding.

## References

[1] Cutler J.A., Mendeloff A.I. (1981). Upper gastrointestinal bleeding. Nature and magnitude of the problem in the U.S. Dig Dis Sci.

[2] Gilbert D.A. (1990). Epidemiology of upper gastrointestinal bleeding. Gastrointest Endosc.

[3] Bajer L., Ryou M., Thompson C.C. (2022). Novel upper-GI bleeding sensor capsule (PILLSENSE): a first in human feasibility and safety. Gastroenterology.

[4] Drastich P., Bajer L., Thompson C.C. (2022). Detection of upper gastrointestinal bleeding using a novel bleeding sensor capsule–a pilot study. Gastrointest Endosc.

